# Circulating lymphocyte is an important determinant of the effectiveness of preoperative radiotherapy in advanced rectal cancer

**DOI:** 10.1186/1471-2407-11-64

**Published:** 2011-02-10

**Authors:** Joji Kitayama, Koji Yasuda, Kazushige Kawai, Eiji Sunami, Hirokazu Nagawa

**Affiliations:** 1 Department of Surgery, Division of Surgical Oncology, University of Tokyo, 7-3-1, Hongo, Bunkyo-ku, Tokyo 113-8655, Japan

## Abstract

**Background:**

Although preoperative radiotherapy (RT) is widely used as the initial treatment for locally advanced rectal cancer (RC) in the neoadjuvant setting, factors determining clinical response have not been adequately defined. In order to find other factors possibly related with radiosensitivity, we evaluated the relationships between circulating blood cell counts and RT effects.

**Methods:**

In 179 cases with advanced RC, we retrospectively examined hemoglobin (Hb) levels and counts of white blood cells (WBC), platelets and WBC subsets before and after RT and investigated their associations with the complete response (CR) rate together with other clinicopathological factors.

**Results:**

The ratio of lymphocytes in WBC taken before RT was significantly greater in 15 CR cases as compared with those in non-CR cases. Patients with high lymphocyte percentages (25.7%) showed better outcome than the counterparts. Conversely, the ratio of neutrophiles was reduced in CR cases. The lymphocyte ratio showed an independent association with CR with multivariate analysis, and tended to be maintained at relatively high levels in CR cases.

**Conclusions:**

In RC patients, peripheral blood lymphocytes have a significant impact on the CR rate in response to RT. Lymphocyte-mediated immune reactions are supposed to have positive roles on clinical response in radiotherapy for RC.

## Background

Previous studies have demonstrated that preoperative radiotherapy (RT) can produce down-staging in advanced rectal cancer (RC), resulting in longer survival, a reduced rate of postoperative local recurrence, and a higher rate of sphincter-preserving surgery. Recently, addition of chemotherapy to RT (CRT) has achieved even more favorable results [1-3], and preoperative RT in the neoadjuvant setting is thus currently recognized as the standard treatment for locally advanced RC. However, in unresponsive cases, RT may have disadvantages such as delaying surgery or immune suppression. Therefore, appropriate selection of patients for preoperative RT is clinically important for improving the outcomes of those with advanced RC.

Previous studies have suggested clinical factors such as the circumferential extent of the tumor, carcinoembryonic antigen (CEA) level, distance from the anal verge [4], temporal pattern of fatigue during CRT [5] and treatment interval between radiation and surgical resection [6] to correlate significantly with clinical response. More recently, radiologic findings such as the maximum standardized uptake value (SUV) for 2-deoxy-2[18F]fluoro-D-glucose (18-FDG)[7] and tumor detection by the microcirculation with dynamic T(1) mapping method using magnetic resonance imaging [8] have been suggested to be useful for response prediction. Biological studies have also indicated the expression levels or patterns of Epidermal Growth Factor Receptor (EGFR) [9,10], Ki-67 [11,12], p21[10], bcl-2/bax [10,11], Vascular Endothelial Growth Factor (VEGF) [12] and thymidine synthetase [10,11] in biopsy specimens to be useful molecular markers. However, controversy persists regarding the results of these prior studies and the data await verification in larger patient populations. Furthermore, treatment outcomes remain difficult to predict due to the lack of appropriate markers predicting clinical responses to CRT.

It was recently suggested that radiosensitivity depends not only on the biological characteristics of tumor cells but also on the tumor microenvironment [13,14]. Since blood cell counts in peripheral blood are considered to reflect environmental host factors in rectal tumor patients, we have endeavored to determine whether the values prior to radiation may serve as new parameters predicting tumor responses to CRT. We also examined the laboratory data during RT and post-RT period before surgery, which may reflect systemic responses against tumor cells damaged by irradiation.

## Methods

### Patients and samples

One hundred and eighty-six (186) patients with rectal adenocarcinoma newly diagnosed received RT between 1997 and 2009 in the Department of Surgical Oncology, Tokyo University Hospital. Among these patients, all pre-RT blood cell counts as well as other clinical and pathological parameters were available in 179 cases, all of whom were included in this study. The 75 patients managed after November 2004 received 5-FU based concurrent chemotherapy (CRT) and the other 104 received RT alone. All values were obtained from patient records. Pre-RT blood data were obtained using samples collected 0-53 days before the start of RT.

Among the 179 patients, 176 underwent total mesorectal excision in the Department of Surgical Oncology. In 12 of the 165 patients, no tumor cells were detected at either the primary site or in regional lymph nodes on pathological examination, confirming pathological complete response (pCR). Three other patients showed a clinical CR (cCR) after CRT, with no detectable cancer cells on multiple biopsy specimens, and were thus followed without surgery. These three patients showed no evidence of recurrence for more than 12 months after completion of RT, and were thus included in the CR group. Informed consent was taken from the patients included in this study and this study was conducted with the approval of the Ethics Committee of the University of Tokyo Hospital.

### Statistical Analysis

The associations of CR with blood cell counts and various other clinical parameters were examined using Wilcoxon's test and the chi-squared test, respectively. Multivariate stepwise logistic regression analysis was performed to determine the independence of all variables identified as possibly significant. All analyses were performed with JMP8.0 software, and p-values less than 0.05 were considered to be statistically significant.

## Results

### Clinical and pathological factors and CR

The clinical or pathological data of the 15 CR and other 164 non-CR cases were shown in Table 1. Node negative cases had a significantly greater CR rate than node positive cases (p < 0.05). The CR rate was higher in patients given CRT than in those receiving RT alone (p = 0.043). In addition, the CR rate tended to be higher in patients with a tumor circumferential extent of less than 60% than in those with values exceeding 60% (p = 0.098). However, none of the other factors, such as tumor size, histological type or CEA level, was significantly associated with the CR rate.

**Table 1:** Clinical and pathological factros in 179 patients with rectal cancer

Variavles	Non-CR (164)	CR (15)	p value
Age	62.7 ± 9.8	64.1 ± 10.9	0.58
Gender			
Male	110	9	0.583
Female	54	6	
T stage			
2	42	4	0.978
3	111	10	
4	11	1	
N stage			
Negative	112	14	0.022*
Positive	52	1	
Clinical stage			
≤ 2	110	13	0.093
3 ≤	54	2	
Histology			
Differentiated	153	14	0.995
Undifferentiated	11	1	
Size			
40 mm <	96	9	0.912
≤ 40 mm	68	6	
Circumferential extent			
60% <	91	5	0.098
≤ 60%	73	10	
Distance from anal verge			
5 cm <	88	6	0.31
≤ 5 cm	76	9	
Chemotherapy			
+	65	10	0.043*
-	99	5	
CEA			
5.0 ng/ml <	90	7	0.526
≤ 5.0 ng/ml	74	8	

*: p < 0.05

### The ratio of lymphocyte and neutrophils were inversely associated with CR rate and survival

**Figure 1:**
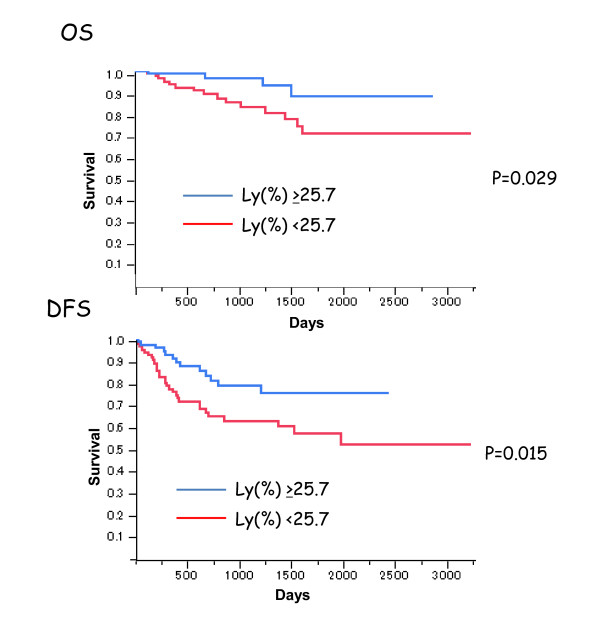
**Overall and disease free survival of the patients with high and low lymphocyte % taken before the RT**. Patients with % of lymphocyte more than 25.7% showed significantly better outcome than those with % of lymphocyte more than 25.7%. The survival curve was constructed with Kaplan-Meier and p value was analyzed with Log rank test.

Blood cell counts taken before RT were compared between CR and non-CR cases (Table 2). Hb levels tended to be higher, while WBC and platelet counts tended to be lower in the CR group, though the differences were not statistically significant. Among WBC subsets, however, the ratio of lymphocytes were significantly greater in CR than in non-CR cases (32.1 ± 9.5% vs 25.6 ± 8.5, p < 0.05). Conversely, the ratios of neutrophils were lower in CR cases (57.8 ± 9.4% vs 64.0 ± 9.5%, p < 0.05). The ratios of eosinophils, basophils as well as monocytes did not show significant difference between CR and non-CR cases. The levels of inflammatory markers, CRP and fibrinogen tended to be lower in CR cases as compared non-CR cases (Table 2). As shown in Table 3 multivariate analysis revealed that lymphocyte ratio as well as N stage showed independent correlation with the CR rate. In addition, when the patients were divided into high and low lymphocyte groups by median ratio of 25.7%, patients with high lymphocyte group showed significantly better outcome in overall and disease free survival (Figure 1)

**Table 2:** Pre-RT blood cell data and clinical and pathological response in rectal cancer patients.

Bloosd Cell data	Non-CR (164)	CR(15)	p value	
Hb	12.8 ± 2.0	13.3 ± 1.1	NS	
WBC	6600 ± 2150	6370 ± 1510	NS	
% neutrophil *	64.0 ± 9.5	57.8 ± 9.4	0.030	
% eosinophil*	3.0 ± 2.8	2.2 ± 1.2	NS	
% basophil *	0.5 ± 0.5	0.6 ± 0.4	NS	
% monocyte *	6.8 ± 2.7	7.3 ± 3.0	NS	
% lymphocyte*	25.6 ± 8.5	32.1 ± 9.5	0.011	
Platelet	27.9 ± 10.9	26.7 ± 8.4	NS	

Each value is the mean ± SD before or after preoperative radiotherapy,

Data were analysed using the Wilcoxon test.

*: Data for white blood cell subsets after radiotheraopy were available in 168

(CR15 and non-CR153) of 179 cases.

**Table 3:** Multivariate analysis for complete response (CR)

Variables	Odds ratio	(95% CI)	p value
N stage: N1 vs N0	0.041	0.034-1.138	0.067
Clinical Stage: 2 vs 3/4	6.864	0.541-9.354	0.193
Circumferential extent: 60%> vs 60% <	0.560	0.396-1.338	0.332
CRT vs RT	2.313	0.855-2.887	0.156
Lymphocytes(%): 25.7% ≤ vs 25.7% >	4.000	1.057-4.455	0.032

Five variables, N stage, Clinical stage, chemotherapy, circumferential extent, % lymphocytes which may have possible correlation with CR rate and their independency were analyzed with stepwise logistic regression analysis using JMP software 8.0.

### Leukocytes subpopulation during RT and post RT period

**Figure 2:**
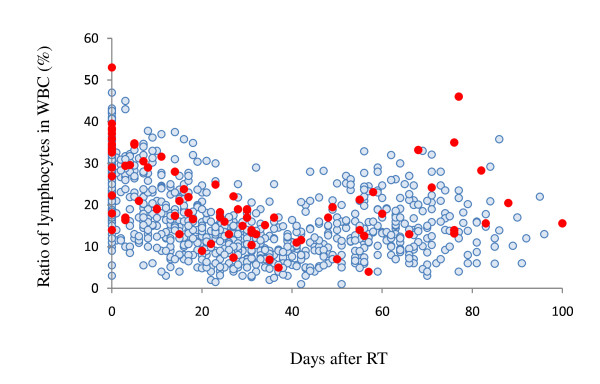
**The change of circulating lymphocytes during RT**. Blood data before the start of RT was set at day 0 and the values obtained at the day from the start of RT were plotted at X axis. The change of lymphocyte percentage in WBC in patient with CR (filled circle) and non-CR (open circle).

Then, we examined the change of the counts of WBC subpopulations during RT and after RT until surgery. Since this is a retrospective study and the timing and frequency of blood tests were largely different among each patient, we plotted the whole data in all of the 179 patients according the days from the initiation of RT. The values of Hb, WBC and platelets tended to be slightly reduced after RT (data not shown). However, the reduction rate was markedly different among WBC subpopulations. Neutrophil counts were relatively stable during the treatment period. In contrast, the number of circulating lymphocytes were markedly reduced during RT and showed gradual increase up to the time of surgery, while monocytes comparatively increased during RT and reduced after RT. When the lymphocyte ratio of the total blood samples were compared between CR and non-CR cases, samples derived from patients of CR group tended to contain more lymphocytes than those from non-CR group (Figure 2). In this retrospective data, the comparison may not have significant meaning from statistical point of view and prospective study is definitely necessary to draw a certain conclusion on this point. However, our data raises a possibility that the circulating lymphocytes may have significant biological effects on tumor response against CRT.

## Discussion

Neoadjuvant radiotherapy (RT) is considered the standard treatment for advanced RC. However, factors predicting the efficacy of neoadjuvant RT, which would be essential for the optimal management of patients with RC, have yet to be fully elucidated. Multiple mechanisms are speculated to contribute to RT-induced antitumor effects[15-17]. In particular, recent studies have suggested that radiosensitivity depends not only on the biological characteristics of tumor cells but also on the tumor microenvironment [13,14]. We, therefore, hypothesized that blood cell counts, which presumably reflect host conditions, may critically affect responsiveness to RT. In fact, our results show clearly that circulating lymphocyte numbers or ratios both before and during RT correlate with the CR rate.

Since the first report in 1979 [18], it has been proposed that tumor shrinkage is not simply dependent on direct damage to irradiated tumor cells, also being greatly affected by the host immune response [19]. In fact, in vivo studies have suggested that cancer cells, dead or dying due to RT and/or chemotherapy, can present tumor-associated antigens to host immune cells and thereby evoke anti-tumor immune responses [20,21]. Moreover, accumulating clinical data suggest the presence of radiation-induced anti-tumor immunity in humans [22,23]. Since lymphocytes, especially T cells, play a central role in anti-tumor immunity, our observation that the percentage of lymphocytes showed a strong association with CR is in accordance with earlier results. In fact, Molling et al demonstrated levels of circulating invariant natural killer T (iNKT) cells to predict the clinical outcomes of patients with head and neck squamous cell carcinoma [24]. It is thus reasonable to speculate that the lymphocyte-mediated immune response against damaged tumor cells is critically important for achieving CR after RT in patients with RC.

In our series, circulating lymphocyte counts decreased drastically after RT, although the reduction was not as pronounced as those in other leukocyte subsets. Peripheral lymphopenia, especially reduced T lymphocytes, after RT was first described in the 1970's [25,26], but the clinical significance of these drops in cell counts has not been well studied. Our extensive search of the literature yielded no previous reports mentioning a significant correlation between circulating lymphocyte counts and RT response, although the degree of recovery of lymphocyte counts after RT reportedly correlates with tumor recurrence in bladder cancer [27]as well as head and neck squamous cell carcinoma [28]. Other reports have suggested that the number of circulating CD4 T lymphocytes is important for the suppression of tumor recurrence [29,30]. These observations allow us to speculate that the radiation-induced depression of lymphocyte counts may provide an opportunity for re-growth via proliferation of tumor cells which survived the irradiation damage, thereby reducing the likelihood of CR after RT. We anticipate that further phenotypic and functional analyses of the characteristics of circulating lymphocyte subpopulations would clarify the mechanisms underlying the responsiveness of tumors to RT.

In contrast to lymphocyte, the neutrophil counts showed inverse correlation with tumor response. Increase of neutrophils counts usually reflects the acute inflammatory response against bacterial infection. In our series, other inflammation markers, such as platelet count, C reactive protein (CRP) and fibrinogen level in serum also showed similar association, although not statistically significant. Previous studies have shown that neutrophils can suppress the T cell response through the production of reactive oxygen species (ROS), nitric oxide (NO) and arginase [31,32]. This may suggest that the presence of acute inflammatory response during CRT causes the suppression of lymphocyte-mediated immunity through the increased circulating neutrophils and thus elicits the unfavorable effects for tumor response.

Circulating lymphocytes number are very prone to affected by various factors such as age, nutrition and chronic stresses. This, in another way, suggest that this value well reflect the total condition of the host to fight with cancer and can be a good marker to tumor response to RT. At least, the lymphocyte ratio of before RT is not largely affected by the timing of blood sampling and thus can be a good prognostic marker. Although the results obtained from this retrospective analysis has a limitation, the significant association between the circulating lymphocyte number and CR rate raises the hypothesis that total eradication of tumor cells after CRT is partially dependent on host immune reaction. Recently, Wichmann et al demonstrated circulating lymphocytes, especially CD4(+) T cells, in patients who received preoperative CTR to be severely decreased in peripheral blood on postoperative days 1 and 2, which may be associated with severe immune dysfunction that actually promotes tumor growth [33]. Lymphocyte-mediated immune reactions are supposed to have positive roles on clinical efficacy of neoadjuvant RT or CRT in the treatment of the patients with advanced RC.

## Conclusion

The number of peripheral blood lymphocytes has a significant impact on radiosensitivity in patients with RC. Lymphocyte-mediated immune reactions may be involved in the clinical response in RT. Augmentation of systemic immunity may improve the effect of neoadjuvant RT for advanced RC.

## Competing interests

The authors declare that they have no competing interests.

## Authors' contributions

JK and KY carried out the all the step of this study. KK and ES participated in the study design and carried out the data analysis including statistics. HN supervised the study. All authors read and approved the final manuscript

## Pre-publication history

The pre-publication history for this paper can be accessed here:

http://www.biomedcentral.com/1471-2407/11/64/prepub
